# A Rare Case of Solitary Fibrous Tumor of the Breast in a Healthy 40-Year-Old Woman

**DOI:** 10.1155/crra/9943282

**Published:** 2025-02-03

**Authors:** Marissa Yaldo, Arif Musa, Michael Aulicino, Brigitte Berryhill

**Affiliations:** ^1^Wayne State University School of Medicine, Detroit, Michigan, USA; ^2^Department of Radiology, Wayne State University/Detroit Medical Center, Detroit, Michigan, USA; ^3^Department of Pathology, Huron Valley Sinai Hospital, Wayne State University/Detroit Medical Center, Detroit, Michigan, USA; ^4^Department of Radiology, Huron Valley Sinai Hospital, Wayne State University/Detroit Medical Center, Detroit, Michigan, USA

**Keywords:** Breast Imaging Reporting and Data System (BI-RADS), mammography, solitary fibrous tumor (SFT), STAT6 stain

## Abstract

Solitary fibrous tumor (SFT) is a rare neoplasm of mesenchymal origin that is primarily found in the lungs but can be found in other locations such as the retroperitoneum, deep soft tissues of the proximal extremities, abdominal cavity, head, and neck. Moreover, SFTs found in the breast are extremely rare and, oftentimes, are found incidentally during screening mammography. Our case presents an exceptionally rare occurrence of a SFT in the breast of a 40-year-old woman. This rarity is underscored by its classification as a SFT and its unusual location within the breast tissue as well as this patient's young age. This case emphasizes the importance of a thorough evaluation with both imaging and histopathology for diagnosing SFTs. It also addresses the potential difficulties that may arise during this process, especially for radiologists who may have limited experience encountering SFTs.

## 1. Introduction

Solitary fibrous tumor (SFT) is a rare neoplasm of mesenchymal origin. This neoplasm is primarily found in the lungs, but extrathoracic locations include the retroperitoneum, deep soft tissues of the proximal extremities, abdominal cavity, head, and neck. Most cases reported to date follow a benign pattern [1]. However, their clinical behavior can be quite unpredictable. Tumors larger than 10 cm in diameter, those with a high mitotic activity rate, and those with histological features such as hypercellularity and necrosis are associated with a significantly increased risk of local recurrence and metastasis [1]. Our case presents an exceptionally rare occurrence of a SFT in the breast. This rarity is underscored by its classification as a SFT and its unusual location within the breast tissue as well as the patient's young age.

## 2. Case Presentation

A 40-year-old female with a palpable left breast lump that she noticed during a self-examination presented for a diagnostic mammogram. She denied any pain or discharge. She also denied any personal or family history of breast cancer or previous breast biopsy but noted her mother previously had lymphoma.

**Figure 1:**
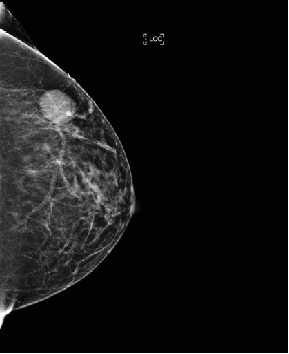
Diagnostic mammogram with craniocaudal view of the left breast showed a smoothly marginated isodense mass measuring approximately 3 cm in the upper outer left breast directly underneath the triangular marker placed over the region of the palpable lump. The breast is comprised of scattered areas of fibroglandular density.

**Figure 2:**
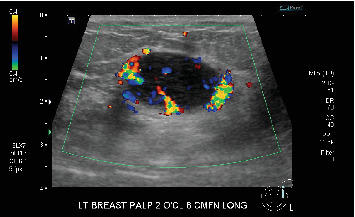
Targeted ultrasound of the left upper outer breast mass demonstrated a smoothly marginated hypoechoic mass 8 cm from the nipple and measuring 2.8 × 1.6 × 2.8 cm with internal and marked color Doppler vascular flow.

Bilateral digital diagnostic mammogram with breast tomosynthesis was performed and showed a smoothly marginated isodense mass measuring about 3 cm in the upper outer quadrant of the left breast (Figure 1). The patient then underwent a targeted breast ultrasound, which confirmed the presence of a 2.8 × 1.6 × 2.8 cm smoothly marginated parallel hypoechoic vascular mass in the upper outer left breast located in the 2:00 position, 8 cm from the nipple (Figure 2).

**Figure 3:**
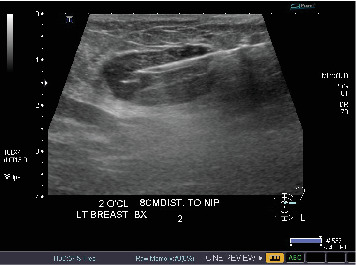
Ultrasound-guided biopsy of the mass in the left upper outer quadrant was performed. The above image shows the biopsy device with a needle tip in the mass.

**Figure 4:**
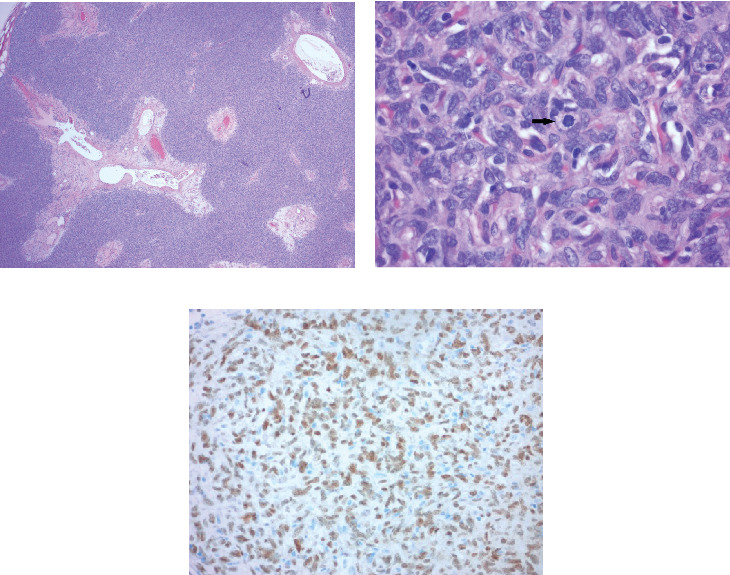
(a) Low-power view demonstrating a solid stromal neoplasm with tumor cells arranged around dilated and branching vasculature (H and E stain, 40× magnification). (b) High-power view showing haphazardly arranged spindle to ovoid cells without significant atypia. Mitoses were evident (arrow) but did not exceed three mitoses per high-powered field (H and E stain, 400× magnification). (c) Immunohistochemistry stain for STAT6 demonstrating strong positivity in tumor cells. AE1/3, desmin, S100 protein, CD31, and ERG were also performed and negative. This phenotype confirms the diagnosis of solitary fibrous tumor (immunohistochemistry stain, 200× magnification).

The finding was assigned the category of 4 according to the Breast Imaging Reporting and Data System (BI-RADS), and ultrasound-guided biopsy was recommended and subsequently performed (Figure 3). Histopathologic findings of the specimen demonstrated spindled to ovoid cells with indistinct cell borders arranged haphazardly around branching and dilated vasculature within the variably collagenous stroma (Figures 4(a) and 4(b)). There was no microscopic evidence of necrosis, hypercellularity, or significant atypia. The mitotic count did not exceed three mitoses per 10 high-powered microscopic fields. Immunohistochemistry (IHC) was performed and the tumor cells were positive for STAT6 (Figure 4(c)) and negative for AE1/3, desmin, S100 protein, CD31, and ERG.

After pathologic consultation, a diagnosis of SFT was ultimately determined, and surgical consultation was recommended for this patient. Subsequently, she underwent a lumpectomy of the left breast. The procedure was well tolerated without intraoperative or postoperative complications.

## 3. Discussion

SFTs are slow-growing neoplasms derived from mesenchymal cells, typically arising in the pleura. SFTs of the breast are rare, and most patients with this neoplasm are diagnosed due to incidental findings on mammography. Most diagnosed cases to date have been found in females, although there have been few reported cases of SFT found in male breast tissue [2]. While most reported cases involve patients in their 50s, the age at presentation has ranged from 49 to 81 years [3]. Unlike the most commonly reported SFTs of the breast in the literature, this case involved a self-identified lump in a patient who had not yet begun her screening mammograms as the patient was only 40 years old at the time of diagnosis.

Although a SFT may present as a well-circumscribed mass without calcification on screening mammogram and as a hypoechoic mass with hypervascularity on ultrasonography, suggestive of a probable indolent course, these lesions must be worked up with further imaging [1, 2]. In fact, the evolving nature of the lesion should be considered, including growth trajectory, and an informed decision should be made prior to proceeding with biopsy. Since many SFTs reported in the literature were initially identified as incidental findings on a screening mammogram, a callback for diagnostic mammogram and adjunctive ultrasound is suggested. Importantly, there is no pathognomonic finding associated with SFT of the breast [1].

Differential diagnoses include fibroadenoma, phyllodes tumor, pseudoangiomatous stromal hyperplasia (PASH), hamartoma, and liposarcoma given that these present as an oval or lobulated well-defined hypoechoic lesion on ultrasound in this population. Unlike fibroadenomas, breast SFTs rarely show calcifications and may exhibit a more homogeneous echo pattern compared to phyllodes tumors, PASH, and hamartomas [2]. Unlike lipomas, hamartomas, and fibroadenomas, which would be expected to exhibit minimal to no color Doppler vascular flow, SFTs are hypervascular lesions, thereby exhibiting increased Doppler flow [2]. Although SFTs are avidly enhancing, the use of contrast to help establish a diagnosis is not well studied and remains an active area of research [4]. Nevertheless, we recommend that the diagnosis of SFT be considered in patients who present with a well-defined mass without calcification on mammography and a hypoechoic, hypervascular mass on ultrasound. Given that the typical SFT exhibits a slow growth pattern, SFT should be considered a more likely diagnosis if the abnormality is slow growing. Subsequent biopsy is necessary to confirm a diagnosis.

A definitive diagnosis requires histopathological analysis and IHC. Histologically, SFTs display an array of disorderly presenting spindle cells with areas of alternating hypercellularity and hypocellularity. Vessels with a branching staghorn appearance may also be appreciated. Tumor necrosis and nuclear pleomorphism are usually absent [3]. However, many of these features overlap with other mesenchymal tumors such as myofibroblastoma, fibroblastic spindle cell tumor, spindle cell lipoma, fibroma, and pseudoangiomatous stromal proliferation [2]. As a result, to differentiate SFTs from other mesenchymal breast lesions, immunohistochemical stains are particularly useful. Typically, SFTs express CD34, Bcl2, CD99, and vimentin, while they do not express actin, desmin, or S100 protein [2]. However, although these IHC stains may help aid in diagnosis, they are not specific for SFT and may exhibit inconsistent expression in some cases.

Notably, the C-terminal part of the STAT6 gene has demonstrated to be a highly specific marker for SFT [5] and, accordingly, was positive in our patient. Although STAT6 positivity alone may not be sufficient to diagnose SFT, the combination of STAT6 positivity and clinical history, pertinent radiologic findings, and histologic studies allows clinicians to establish a firm diagnosis of SFT.

This patient underwent lumpectomy in accordance with the gold standard of management for SFTs, which involves resection of the tumor with clear surgical margins for both benign and malignant SFTs of the breast. Typically, SFTs of the breast do not recur, and to date, there are only two cases of breast SFT recurrence in the literature [6]. Therefore, there are currently no consensus guidelines for surveillance following gold standard treatment. However, patients with risk factors such as age greater than 55 years or patients with tumors featuring high mitotic activity and/or size greater than 15 cm may need closer surveillance due to increased risk of recurrence. Surveillance guidelines based on findings from other breast tumors recommended frequency of surveillance every 3 months for the first 2 years after surgery, every 6 months for years 2–5 after surgery, and then at 5 years after the surgery [5]. As a result, we recommend that patients diagnosed with SFT of the breast and exhibiting high-risk factors, as discussed above, be followed up with regular surveillance given the potential unpredictability in the clinical course of these tumors [1].

## 4. Conclusion

SFTs of the breast are exceedingly rare. Due to their infrequent incidence and varied histological appearances, they can pose challenges in diagnosis and management, particularly for radiologists who may not encounter them frequently. Additionally, imaging features of SFT may overlap with those of other mesenchymal breast lesions, making accurate diagnosis challenging. It is crucial to be aware of any slow-growing lesion of the breast and consider multimodal imaging techniques and histopathological examination for definitive diagnosis and appropriate management. A STAT6 IHC stain should be included in the histopathological workup as positivity may aid in establishing a firm diagnosis.

## Data Availability

The data supporting the findings of this case report, including radiological images and clinical details, are available within the article. Inquiries regarding additional data can be made available by the corresponding author.
